# Critical medical ecology and SARS-COV-2 in the urban environment: a pragmatic, dynamic approach to explaining and planning for research and practice

**DOI:** 10.1186/s40249-020-00694-3

**Published:** 2020-06-19

**Authors:** Timothy De Ver Dye, Erin Muir, Lorne Farovitch, Shazia Siddiqi, Saloni Sharma

**Affiliations:** Department of Obstetrics and Gynecology, University of Rochester School of Medicine and Dentistry, 601 Elmwood Avenue, Rochester, New York 14642 USA

## Abstract

**Background:**

Practitioners and researchers in the midst of overwhelming coronavirus disease 2019 (COVID-19) outbreaks are calling for new ways of looking at such pandemics, with an emphasis on human behavior and holistic considerations. Viral outbreaks are characterized by socio-behaviorally-oriented public health efforts aimed at reducing exposure and prevention of morbidity/mortality once infected. These efforts involve different points-of-view, generally, than do those aimed to understand the virus’ natural history. Rampant spread of SARS-CoV-2 infection in cities clearly signals that urban areas contain conditions favorable for rapid transmission of the virus.

**Main text:**

The Critical Medical Ecology model is a multidimensional, multilevel way of viewing pandemics comprehensively, rooted simultaneously in microbiology and in anthropology, with shared priority for evolution, context, stressors, homeostasis, adaptation, and power relationships. Viewing COVID-19 with a Critical Medical Ecological lens suggests three important interpretations: 1) COVID-19 is equally — if not more — a socially-driven disease as much as a biomedical disease, 2) the present interventions available for primary prevention of transmission are social and behavioral interventions, and 3) wide variation in COVID-19 hospitalization/death rates is not expected to significantly be attributable to a more virulent and rapidly-evolving virus, but rather to differences in social and behavioral factors — and power dynamics — rather than (solely) biological and clinical factors. Cities especially are challenged due to logistics and volume of patients, and lack of access to sustaining products and services for many residents living in isolation.

**Conclusions:**

In the end, SARS-CoV-2 is acting upon dynamic social human beings, entangled within structures and relationships that include but extend far beyond their cells, and in fact beyond their own individual behavior. As a comprehensive way of thinking, the Critical Medical Ecology model helps identify these elements and dynamics in the context of ecological processes that create, shape, and sustain people in their multidimensional, intersecting environments.

## Background

As the novel severe acute respiratory syndrome coronavirus 2 (SARS-CoV-2; the cause of the coronavirus disease 2019 [COVID-19]) is transmitted globally to often devastating circumstances for individuals, communities, and countries, researchers seek to identify how to contribute global scientific insights by including this new experience into their research [1, 2]. In many ways, researchers have been primed for this moment for quite some time: especially over the past decade, there has been a push to create multi- and cross-disciplinary work, global collaboration, and integrative models to help address complex problems [3]. Such models can serve as useful heuristic devices that add perspective and points-of-view that contribute to the overall scientific canon aimed at explaining, reducing, and preventing health crises.

Practitioners and researchers in the midst of overwhelming COVID-19 outbreaks are calling for new ways of looking at such pandemics, with an emphasis on human behavior and holistic consideration [4]. While, in effect, any scientific model could be used to frame SARS-CoV-2 and COVID-19 to generate hypotheses and insights, some models are especially well-suited to thinking about pandemics and to expand comprehensive thinking beyond traditional — albeit useful — ways of looking at viral outbreaks. Pandemics are frequently characterized by intense focus on the evolution, transmission, survival, and pathogenic impact of the virus itself — indeed development of treatments and immunizations rely on understanding these biological elements. Viral outbreaks, however, are also characterized by more socio-behaviorally-oriented public health efforts aimed at reducing and eliminating exposure to the virus and prevention of morbidity and mortality once infected [5, 6]. These public health and behavioral efforts involve different forms of science and points-of-view, generally, than do those efforts attempting to understand the virus’ natural history. Often missing from scientific thinking around pandemics are the multi-leveled, complex notions surrounding social and cultural determinants of health and behavior, which focus beyond the individual and their cells to systematic and structural elements in society that additionally contribute to disease and its spread. Engulfing social determinants are power and economic relationships and processes, at micro and macro levels, that subsequently realign causal attention from individuals as the pandemic’s focus of control to larger population aggregations. All of these intersecting dimensions are overlapping, dynamic, and changing. But, whether or not an individual person dies from COVID-19 relates — in part — to them being exposed in the first place (a largely social-environmental phenomenon), the voracity of the virus pitted against one’s cells (a largely bio-pathogenic process), and the ability for that pathogenic process to be interrupted or attenuated (a mix of social and biological processes, including health care systems and practices).

**Fig. 1:**
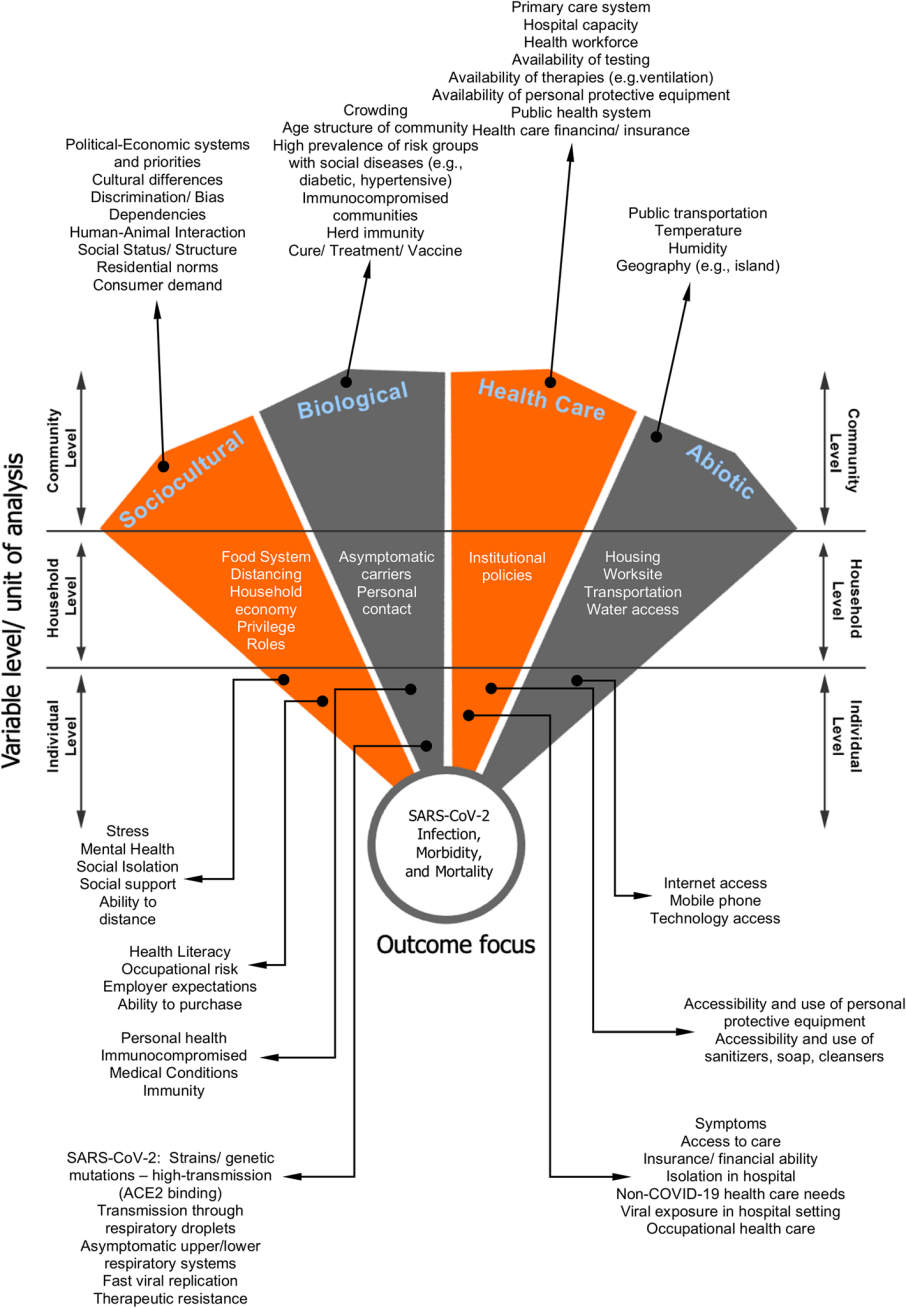
Critical Medical Ecological Model Applied to SARS-CoV-2 infection and COVID-19 (adapted from McElfroy and Townsend 2015). SARS-CoV-2: Severe acute respiratory syndrome coronavirus 2; COVID-19: Coronavirus disease 2019; ACE2: Angiotensin-converting enzyme 2

The peculiar, intersectional social and biological historical circumstances that bring virus and human together are crucial aspects of explaining viral infection in the context of these other dimensions. One especially well-suited paradigm for thinking about SARS-CoV-2 and COVID-19 in this manner is the Critical Medical Ecology model [7–10]. Distinct from other ways of integrative thinking (perhaps the Biopsychosocial Model, [11] or the Social Ecological Model [12]), Critical Medical Ecology is a multidimensional, multilevel way of viewing pandemics comprehensively, rooted simultaneously in microbiology and in anthropology, with shared priority for the processes of evolution, context, unintended/unexpected consequences, stressors, homeostasis, adaptation, and power relationships — the main components of ecology, with attention to power dynamics that have widespread consequence [10]. The spread and impact of SARS-CoV-2/ COVID-19 differentially impacts cities; in fact, the nodes of the pandemic are cities, which can be viewed as similarly evolving, reacting, and adapting as entities like pathogens and humans [13]. The Critical Medical Ecology model — with its emphasis on multileveled factors from cell to society and experiences across sociocultural, biological, abiotic, and health care domains (Fig. 1) — is well-suited to, therefore, help conceptualize (as an *explanatory* model) or to help plan for (as a *programmatic* model) the factors, domains, and processes that surround SARS-CoV-2, COVID-19, and urban areas.

### The virus in ecological context

Crucial features of the biology of SARS-CoV-2 are the central pathogenic stressors in the COVID-19 experience. Coronaviruses comprise an enveloped, single positive-strand RNA virus with spikes [14] that are essential for viral entry into cells and that enable replication of the single strand RNA via RNA-dependent-RNA polymerase in the infected cytoplasm of the cell [14]. At least eight strains of SARS-CoV-2 circulate around the world, giving clues to complex transmission patterns [15]. For example, the L-type became more prevalent than the S-type strain of SARS-COV-2 after being declared the global pandemic, suggesting that SARS-CoV-2 virus perhaps evolved and may create different outcomes [16]. Most COVID-19 cases are mild or asymptomatic; those individuals with no symptoms, however, can transmit the virus, a key feature of the biology of SARS-CoV-2 infection [17]. The peak of viral load concentration is estimated at approximately Day 5 of symptom onset with viral shedding through respiratory droplets or fecal matter. That those infected with SARS-CoV-2 might be contagious from a few days before symptoms begin to approximately 20 days after symptoms appear [18] drives the primary preventive strategy of physical isolation as an attempt to stop or slow community transmission.

Transmission of SARS-CoV-2 arises from the intersection of biology with human behavior, culture, and the abiotic environment. The natural host for SARS-CoV-2 is likely to be bats. Most coronaviruses are found in regions with high diversity of bats, showing that coronaviruses have the capability to inhabit various species of bats, with high prevalence of host — an important evolutionary factor in emerging infectious disease [19]. The intermediate host for SARS-CoV-2 is suspected to be pangolin, one of the world’s most illegally-trafficked animals and listed on the International Union for Conservation of Nature (ICUN) Red List of Threatened Species [20]. While no data of seasonality for coronaviruses has yet been established, significant difference in the growth rate of SARS-CoV-2 infection exists between cold and warm climates [21]. Based on the cases from January 22, 2020 to March 21, 2020, there were fewer cases of the virus in warmer climates with more cases of the virus in areas within the range of temperature (3–17 °C) and absolute humidity (4 to 9 g/m^3^) [21].

The transmission rate of SARS-COV-2 is very high, possibly due to the capability of infection in both upper and lower respiratory systems and through several mechanisms of pathogenesis (e.g., angiotensin-converting enzyme 2 [ACE2], polybasic furin-type cleavage site, and antibody-dependent enhancement) [22]. Also, SARS-COV-2 has higher binding affinity toward ACE2 receptors comparing with SARS-CoV, which suggests that SARS-CoV-2 is more contagious [23]. COVID-19 is more common in people with diseases and behaviors that are strongly sociocultural: immunocompromised individuals, those with pre-existing chronic conditions (hypertension, diabetes), smokers, and elders [24].

### Cities in ecological context

Rampant spread of SARS-CoV-2 infection within and among cities clearly signals that urban areas contain conditions favorable for rapid transmission of the virus. As social-environmental entities, cities evolved historically to support the proximity of services and economic specialization required to sustain growing populations and dramatic shifts in agricultural production [13]. Cities emerged as a new built environment created and shaped by the people within it, and subsequently shaping them in return [13]. Higher concentrations of people require that cities create institutions and infrastructures supporting education, jobs, housing, healthcare and transportation, all organized around the presumed requirement of serving large volumes of people dependent upon external provision of services. Life in an urban environment presents opportunities for access to technology, employment, and better healthcare, but also exposes limitations, underscored by living in large, unmanageable, and often inequitable aggregations of people, frequently in small residences in limited geographical space.

While cities typically serve (or are perceived as serving) as resource-rich economic, transportation, and cultural hubs, the challenges of public health, medical care, and disease in urban areas are accentuated in the global COVID-19 pandemic. In the absence of a cure or vaccine against SARS-CoV-2, isolation, quarantine, limiting community interaction, providing personal protective equipment for front-line providers, and social-physical distancing are the primary strategies available to interrupt or attenuate viral transmission [25]. The current predictive models for SARS-CoV-2 infection [25] and genomic analysis of the virus from different regions of the world [15] show that travelers from affected areas are the first points of contact for spreading infection before community transmission ensues. Viral transmission is further enabled in cities by the complex interaction of high population density, a heterogenous population, mass public transit systems such as buses and subways, unequal exposure to risk and virus, and also a higher rate of nosocomial transmission through over-crowded hospitals and healthcare facilities [26]. The current “shelter in place” orders issued by governments are an almost unimaginable premise in metropolitan cities in that essential services supply chains (e.g., food, protective gear) can be disrupted or manipulated by differential purchasing power in the face of urban inequality, especially exacerbating the vulnerability of marginalized groups (including people living with disabilities, immigrants, and homeless) who frequently are not able to adhere to preventative guidelines [27].

City life and urbanization therefore present a unique challenge for global pandemic containment, as viruses are likely to arrive in cities first due to their global connectivity, and are more likely to thrive in their complex artificial ecosystems. Cities are the first and most substantial abiotic environments where SARS-CoV-2 arises, with public areas, bars, schools, hospitals, and workplaces providing ideal conditions for the virus to spread [28]. While access to enhanced technology and resources potentially allows for expedited short-term adaptation of people and systems, inequities bar many from penetrating basic health, social, and sustenance services as urban systems quickly become overwhelmed by the large volume of people in need.

### Health care in ecological context

Health, medical, and healing systems are especially relevant cultural adaptations that have evolved over time; an evolution that was, in fact, often in tandem with human cultural responses to infectious disease [29]. Reduction in the morbidity and mortality from SARS-CoV-2 infection requires a prevention system sensitive to the biological features of the virus previously described, in particular its easily aerosolized transmissibility, contagious symptomatic and asymptomatic carriers, and, in some, rapid progression from symptoms to systemic medical crisis. No curative treatment (tertiary prevention) exists for COVID-19, underscoring the need for screening and primary/secondary prevention with subsequent public health intervention. Identifying SARS-CoV-2 infection comprises symptomatic screening, testing for presence of viral DNA (PCR), or a serological test for presence of viral antibodies. Given the large burden of COVID-19 attributable to viral transmission by asymptomatic carriers, widespread testing and subsequent quarantine of those found positive is necessary to interrupt transmission of infection. Major challenges to symptomatic screening for COVID-19 are the ambiguities and similarities of symptoms compared to the common cold or influenza [30]. Widespread and timely testing varies greatly from country to country, and in the USA especially (where testing policy is not regulated nationally, and instead becomes the responsibility of local entities), from community to community local officials compete with others within their own state and across states to access supplies through undisclosed sources [31]. The centralized push for testing in the USA by Centers for Disease Control and Prevention was mired in bureaucracy, manufacturing issues, quality control, and scientific standard delays [32]. WHO shipped 250 000 diagnostic tests to 70 laboratories around the world while the US efforts in developing testing lagged behind [33]. Republic of Korea was able to offer testing kits after China shared the genomic sequence with the public and emergency approval was granted on a temporary basis [34]. The medical teams of Korea were able to screen patients with suspected respiratory symptoms in a time-efficient manner by donning one quarantine suit in “drive-through centers” that minimized contact between health care workers and patients [34].

Because of the voracity and lack of experience with SARS-CoV-2, treatments are taking time to emerge and best-practice remains unclear. The multipronged approach to treatment includes development of pharmaceutical regimens, and (for the most severely impacted patients) the use of life-saving equipment (such as ventilators) to treat symptoms, all requiring rapid expansion in hospital beds and capacity. Social-physical distancing are the primary strategies available to interrupt or attenuate viral transmission [35], and some of preventive measures (such as hand hygiene or wearing a face mask) were found to reduce levels of psychological impact during COVID-19 outbreak and peak of the epidemics [35, 36].

Finally, providing protective gear and preventive policies for the healthcare workforce — among those at greatest risk for SARS-CoV-2 infection — has lagged behind the rapidly rising numbers of cases of COVID-19 cases and raised the stress levels of healthcare workers [37]. Delays in production and deployment of life-saving equipment for patients and care providers have been influenced by political and economic priorities well-beyond the patient-provider interface and the institutions where these activities occur [38–40].

## Conclusions

Viewing COVID-19 with a Critical Medical Ecological lens suggests three important interpretations: 1) COVID-19 is equally — if not more — a socially-driven disease as much as a biomedical disease, 2) the present interventions available for primary prevention of transmission are social and behavioral interventions, and 3) power dynamics and political relationships well beyond the level of the individual likely determine, at least in part, the risk that a person will become infected. Human sociocultural factors (e.g., consumption of pangolins, travel exposures, face-to-face interaction) and socially-involved predisposing conditions (hypertension, diabetes, smoking) provide a platform for this virus to spread. Many — if not most — deadly viruses circulating throughout the world arose from the human-animal interface, challenging agricultural and dietary practice, disease effects of modernity, and culture. Class privilege contributes to some groups willfully disregarding social-physical distancing orders while disadvantaging others who serve them in restaurants, stores, and delivery services. The healthcare workforce at the forefront of caring for those infected are at-risk for contracting SARS-CoV-2 because of shortages of personal protective equipment (attributable to differential access to supply chain by many actors) as are workers considered “essential” throughout the service industy similarly without access to protective gear [41]. Patients are differentially disadvantaged by lack of universal screening and testing policies and the supply chain of tests to support them, combined with unequal access to equipment to treat the most serious cases. Supply chains across the spectrum of testing and treatment are subject to competition and privileged access depending on political and economic relationships. Cities especially are challenged due to logistics and volume of patients, and lack of access to sustaining products and services for many residents living in isolation. What happens to people is largely a function of who and where they are. Wide variation in hospitalization rates and death rates from COVID-19 are not expected to be attributable significantly to a more virulent and rapidly-evolving virus, but to social and behavioral factors — and power dynamics — rather than (solely) biological and clinical factors. In the end, SARS-CoV-2 is acting upon dynamic social human beings, entangled within structures and relationships that include but extend far beyond their cells, and in fact beyond their own individual behavior. As a comprehensive way of thinking, the Critical Medical Ecology model helps identify these elements and intersections in the context of ecological processes that create, shape, and sustain people in their multidimensional, intersecting environments.

## Data Availability

Not applicable.

## Abbreviations

SARS-CoV-2: Severe acute respiratory syndrome coronavirus 2
COVID-19: Coronavirus disease 2019
WHO: World Health Organization
ACE2: Angiotensin-converting enzyme 2
